# High Abundance of Proteobacteria in Ileo-Anal Pouch Anastomosis and Increased Abundance of Fusobacteria Associated with Increased Pouch Inflammation

**DOI:** 10.3390/antibiotics9050237

**Published:** 2020-05-08

**Authors:** Andreas Munk Petersen, Hengameh Chloé Mirsepasi-Lauridsen, Marianne K. Vester-Andersen, Nikolaj Sørensen, Karen Angeliki Krogfelt, Flemming Bendtsen

**Affiliations:** 1 Gastrounit, Medical Section, Amager-Hvidovre University Hospital, 2650 Copenhagen, Denmark; marianne@kajbaek.dk (M.K.V.-A.); Flemming.Bendtsen@regionh.dk (F.B.); 2 Department of Clinical Microbiology, Amager-Hvidovre University Hospital, 2650 Copenhagen, Denmark; 3 Faculty of Health and Medical Sciences, University of Copenhagen, 2100 Copenhagen, Denmark; 4 Department of Bacteria, Parasites and Fungi, Statens Serum Institut, 2300 Copenhagen, Denmark; pas@ssi.dk (H.C.M.-L.); KAK@ssi.dk (K.A.K.); 5 Department of Internal Medicine, Zealand University Hospital, 4600 Køge, Denmark; 6 Clinical-Microbiomics, Ole Maaløes Vej 3, Clinical Microbiomics, 2200 Copenhagen, Denmark; Nikolaj@clinical-microbiomics.com; 7 Department of Virus and Microbiological Special Diagnostics, Statens Serum Institut, 2300 Copenhagen, Denmark; 8 Department of Science and Environment, Roskilde University, 4000 Roskilde, Denmark

**Keywords:** Proteobacteria, *Escherichia coli*, Fusobacteria, pouchitis, inflammatory bowel disease, calprotectin

## Abstract

Low diversity intestinal dysbiosis has been associated with inflammatory bowel disease, including patients with ulcerative colitis with an ileo-anal pouch anastomosis. Furthermore, specific *Escherichia coli* phylogroups have been linked to inflammatory bowel disease. Our aim was to characterize the differences among microbiota and *E. coli* phylogroups in active and inactive pouchitis. Disease activity was assessed using the modified pouch disease activity index and by fecal calprotectin. Microbiota diversity was assessed by 16S rDNA MiSeq sequencing. *E. coli* phylogroup was determined after triplex PCR. Twenty patients with ulcerative colitis with an ileo-anal pouch anastomosis were included, 10 of whom had active pouchitis. Ileo-anal pouch anastomosis patients had an increased abundance of Proteobacteria colonization compared to patients with ulcerative colitis or Crohn’s disease and healthy controls, *p* = 1.4·10^−5^. No differences in *E. coli* phylogroup colonization could be determined between cases of active and inactive disease. No significant link was found between α-diversity and pouch inflammation. However, higher levels of Fusobacteria colonization were found in patients with a pouch with a fecal calprotectin level above 500, *p* = 0.02. In conclusion, patients with a pouch had an increased Proteobacteria abundance, but only Fusobacteria abundance was linked to inflammation.

## 1. Introduction

Some patients with ulcerative colitis (UC) will experience an extensive flare-up of colonic inflammation that does not respond to treatment with 5-ASA, prednisolone, anti-inflammatory drugs or to biological treatments (e.g., TNF-α inhibitors). In these cases, approximately 15% of all patients with UC, a proctocolectomy is often required [1]. Patients will most often subsequently be given the option to have an ileo-anal pouch-anastomosis (IAPA) performed. Patients with UC and with an IAPA will quite frequently (incidence ranges from 14% to 59%) experience episodes of inflammation in their pouch [2]. This is in stark contrast to patients who have undergone colectomy and IAPA due to familial colonic polyposis, who rarely (0–11%) experience pouchitis [3]. Interestingly, the most efficient treatment of pouchitis is antibiotic treatment, e.g., with metronidazole or ciprofloxacin, whereas traditional treatment of UC, in the form of immunomodulators, seems less efficient [4]. 

It has previously been documented that patients with UC can benefit to varying degrees from antibiotic treatments [5]. Furthermore, recent data indicate that remodulation of the gut microbiota in the form of fecal microbiota transplantation (FMT) will reduce intestinal inflammation in at least some patients with UC [6]. These circumstances suggest that studies of IAPA pathogenesis could also inform our understanding of inflammatory bowel disease (IBD) pathogenicity in general. UC, Crohn’s disease (CD) and pouchitis have been linked to changes in the intestinal microbiota (dysbiosis) [7], but whether this altered microbiome in IBD is a cause or an effect of the intestinal inflammation, has yet to be determined [8]. The clinical effect of both antibiotics and FMT does, however, seem to provide some support for a theoretical causative role of intestinal microbiota changes. This could be due to either an infection with a thus far unrecognized pathogen, or through a malfunction of the intestinal homeostasis brought about by intestinal low diversity dysbiosis. 

One of the most important findings in CD and UC has been increased Proteobacteria in these patients when compared to healthy controls [9,10,11], and in this context, links have been found between a specific *Escherichia coli* phylogroup, phylogroup B2, and an active inflammation in UC and CD [12]. Whether these changes in *E. coli* phylogroup can also be found in patients with pouchitis remains unknown. Pouchitis is basically diagnosed by scoring of symptoms and endoscopic appearance [13]. In daily practice, a simple clinical version of the modified pouchitis disease activity index (mPDAI) [14] is often used instead of endoscopic evaluation, which patients find unpleasant and sometimes painful. The purpose of our study was to evaluate fecal microbiota changes and changes in *E. coli* clonality associated with active pouchitis in patients with known recurrent pouchitis. 

## 2. Results

### 2.1. Patients Characteristics

A total of 20 patients (ten male and ten female), median age 40 (24 to 79), with IAPA were included in this study (Table 1). Ten of these had a mPDAI score above or equal to 3, and 10 had a score below 3. A significantly elevated fecal calprotectin above 500 was found in six patients, two of these with a mPDAI below 3. Patients with a mPDAI of 3 or above, compatible with active pouchitis, had a median fecal calprotectin of 204 (IQR, 88-1418); patients with a mPDAI below 3 had a median fecal calprotectin of 119 (IQR, <50–260) (Table 1). 

### 2.2. OTU and Shannon Index

**Figure 1:**
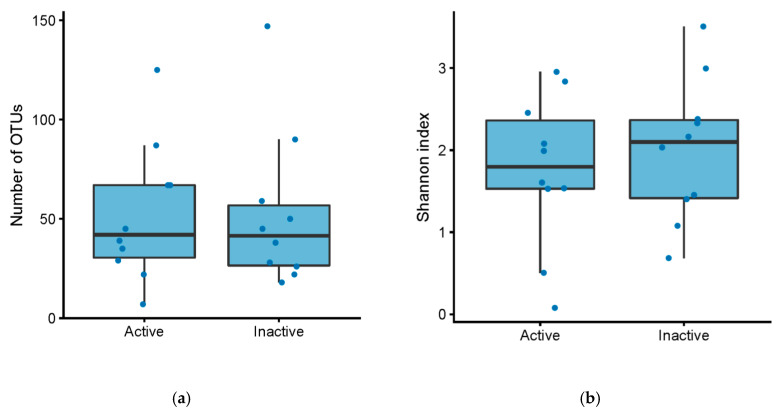
(**a**) Number of OTUs in patients with an ileo-anal pouch anastomosis, with active vs. inactive pouchitis. Active pouchitis was defined as a modified pouchitis disease activity index (mPDAI) score of 3 or above. (**b**) Shannon diversity index in patients with an ileo-anal pouch anastomosis, with active vs. inactive pouchitis. Active pouchitis was defined as a mPDAI score of 3 or above.

**Figure 2:**
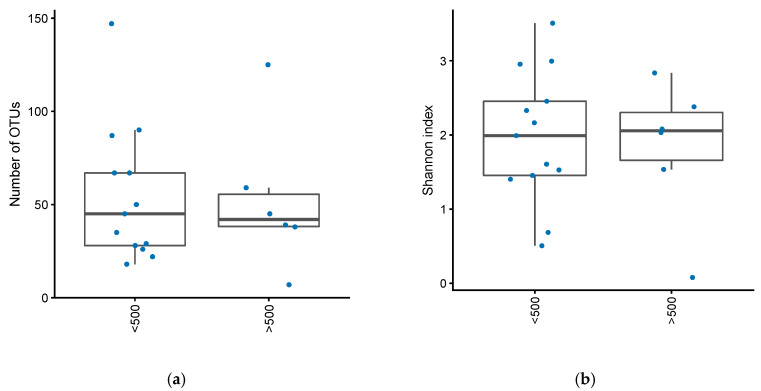
(**a**) Number of OTUs in patients with an ileo-anal pouch anastomosis, with or without increased inflammation in their pouch. Increased inflammation defined as fecal calprotectin greater than 500. (**b**) Shannon diversity index in patients with an ileo-anal pouch anastomosis, with or without increased inflammation in their pouch. Increased inflammation was defined as a fecal calprotectin greater than 500.

There was no difference in the number of operational taxonomical units (OTUs) between active and inactive pouch inflammation, based on a mPDAI of 3 or higher (Mann–Whitney U test, *p* = 0.84) or fecal calprotectin greater than 500 (Mann–Whitney U test, *p* = 0.98) (Figure 1a, Figure 2a). Similarly, there was no significant difference in Shannon index, basing activity on a mPDAI of 3 or higher (Mann–Whitney U test, *p* = 0.74) (Figure 1b) or fecal calprotectin greater than 500 (Mann–Whitney U test, *p* = 0.97) (Figure 2b).

**Figure 3:**
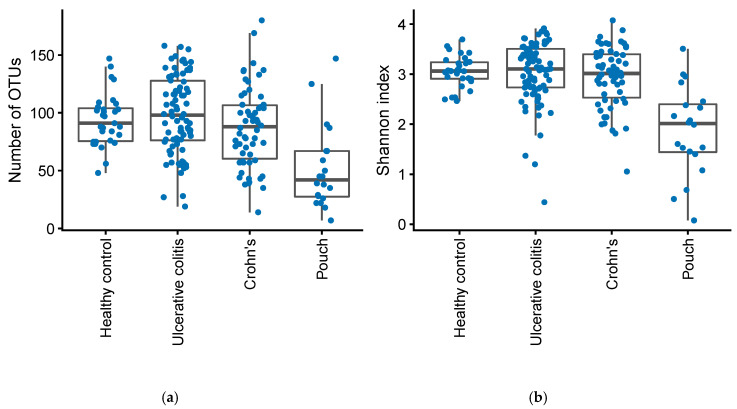
Number of OTUs (**a**) and Shannon diversity index (**b**) in patients with Inflammatory Bowel Disease (Crohn’s disease, N = 58 and Ulcerative Colitis, N = 82), controls, N = 31, and in patients with an ileo-anal pouch anastomosis, N = 20.

Compared to healthy controls and patients with UC and CD, patients with a pouch had significantly fewer OTUs (Mann–Whitney U test, *p* = 3.4·10^−6^) and a lower Shannon index (Mann–Whitney U test, *p* = 8.8·10^−8^), Figure 3. 

### 2.3. Bacterial Composition

**Figure 4:**
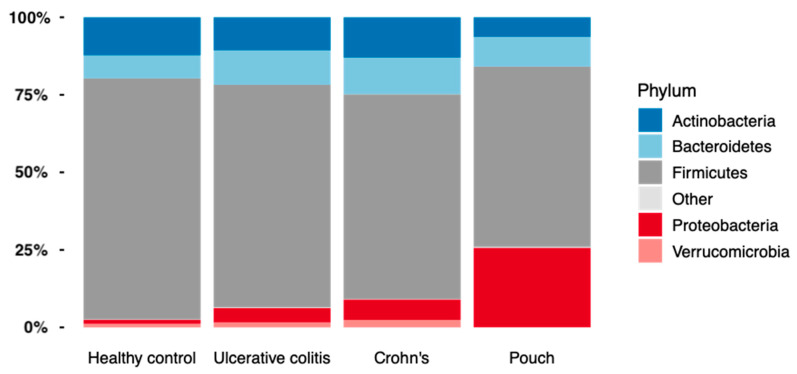
The relative abundance of bacterial phyla found in Inflammatory Bowel Disease (Crohn’s disease, N = 58 and Ulcerative colitis, N = 82), controls, N = 30, and in patients with a pouch, N = 20.

**Figure 5:**
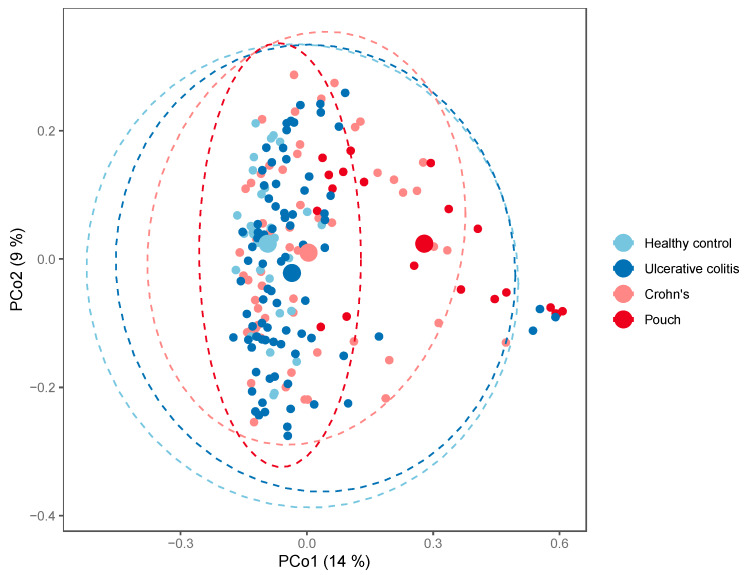
PCoA plot of generalized UniFrac distances by patient group; Inflammatory Bowel Disease (Crohn’s disease, N = 58 and Ulcerative colitis, N = 82), controls, N = 30, and in patients with a pouch, N = 20. The small dots are samples, while the big dots are centroids of their respective groups.

When looking at bacterial phyla with a prevalence of >20%, patients with a pouch had a remarkable higher proportion of Proteobacteria compared with the other groups (Mann–Whitney U test, *p* = 1.4·10^−5^, FDR = 8.43·10^−5^), while the abundance of Actinobacteria, Bacteroidetes, and Verrucomicrobia was significantly lower in patients with a pouch (Mann–Whitney U tests, FDR < 0.10), Figure 4. A similar analysis of genera found 57 out of 75 genera to be significantly different in abundance between patients with a pouch and the other three groups (Mann–Whitney U tests, FDR < 0.10). When using generalized UniFrac distances [15], the microbiome separated significantly by patient group (permutational MANOVA, R2 = 6.7%, *p* = 0.001), patients with a pouch especially having a distinct microbiome (Figure 5).

**Figure 6:**
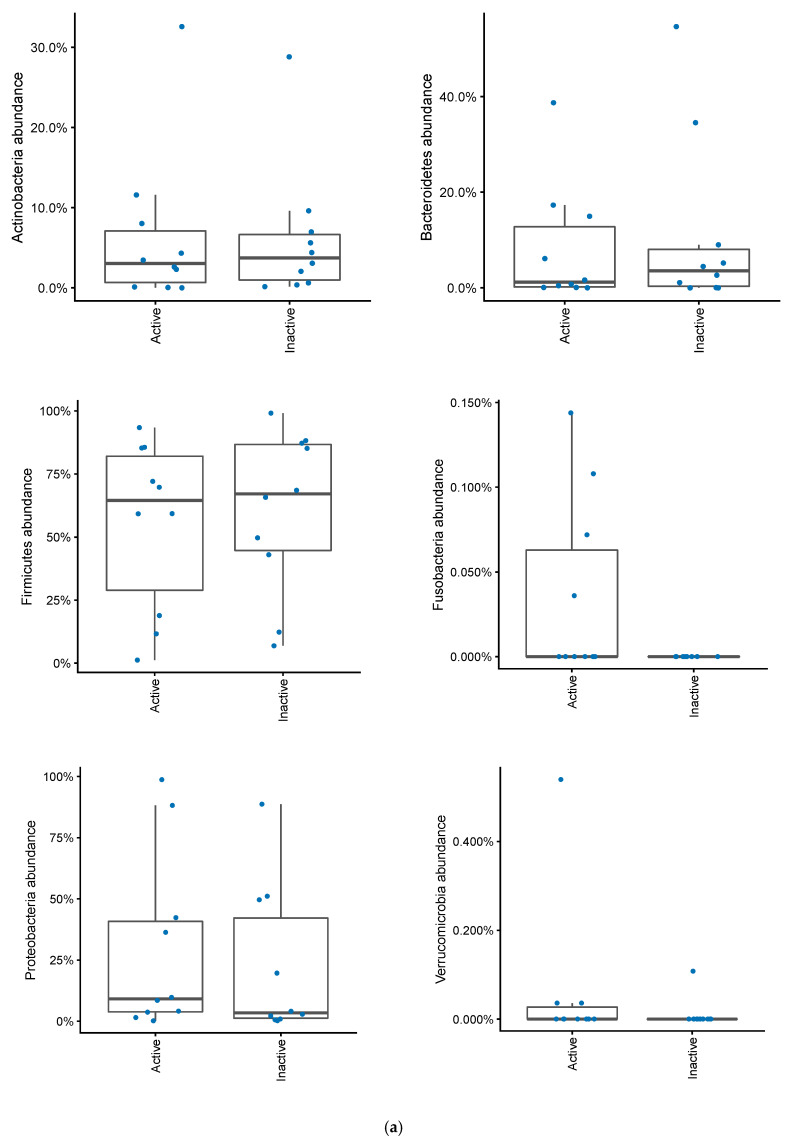

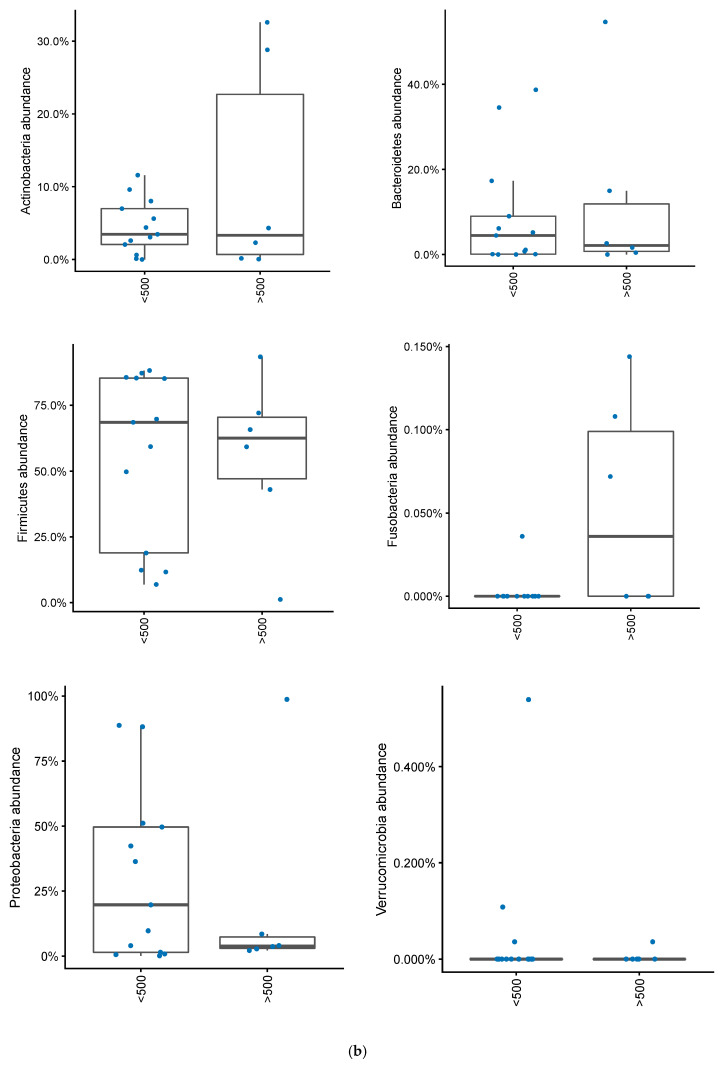
(**a**) Changes in bacterial phyla in patients with an ileo-anal pouch anastomosis, with or without active pouchitis. Pouchits defined as as a mPDAI score of 3 or above. (**b**) Changes in bacterial phyla abundances in patients with an ileo-anal pouch anastomosis, with or without increased inflammation in their pouch. Increased inflammation was defined as a fecal calprotectin greater than 500.

In patients with a pouch, disease activity based on mPDAI was not significantly associated with a differential abundance of Actinobacteria, Bacteroidetes, Firmicutes, Proteobacteria or Verrucomicrobia (Figure 6a). There was a tendency for higher levels of Fusobacteria in patients with a mPDAI of 3 or higher (Mann–Whitney U test, *p* = 0.09). When looking at patients with fecal calprotectin greater than 500, Fusobacteria were significantly more abundant (Mann–Whitney U test, *p* = 0.02), without any significant differences among the other bacterial phyla (Figure 6b). Correlating calprotectin and mPDAI with genus and phylum abundances (of taxa with >20% prevalence) gave no significant results (Spearman correlations, FDR > 0.10).

### 2.4. E. coli Phylogenetic Groups

No associations with *E. coli* phylogenetic groups (A, B1, B2, D) in samples could be found regarding activity, whether activity was defined according to mPDAI or fecal calprotectin (Table 1). Overall, two of the ten patients with active pouchitis based on their mPDAI were colonized with phylogroup B2 *E. coli,* compared to three of the ten patients without pouchitis. Likewise, two of the six patients with fecal calprotectin above 500 were colonized with phylogroup B2 *E. coli*, compared to three of the 14 patients with fecal calprotectin below 500. None of these differences were statistically significant.

## 3. Discussion

Based on phyla and genus level community compositions and alpha and beta diversity, it is apparent that patients with a pouch have a microbiome very distinct from that of healthy controls, but also from patients with UC and CD. However, in this study, no statistically significant changes in intestinal microbiota were found between active and inactive pouchitis based on patient symptom scores. Microbiota differences between patients with a pouch with and without pouchitis have previously been described as minimal. A recent study found a decrease in the relative abundance of Bacteroidetes when stool frequency was increased, but at the same time found no bacterial taxa to be associated with the overall clinical activity score [16]. In a review of studies comparing microbiota in pouchitis versus healthy pouches, an increase of *Clostridium* species was suggested as being linked to pouchitis [17], even though it was concluded that inconsistent findings across studies mean that it is difficult to posit a causative relationship between these changes and pouchitis.

Interestingly, we did find a tendency towards a greater abundance of Fusobacteria among patients with the highest symptom scores. This association was even stronger when looking specifically at the patients with the highest inflammatory burden, i.e., with a fecal calprotectin above 500, suggesting that Fusobacteria might contribute to the inflammatory process in pouchitis. Even though we did not find a positive correlation with mPDAI or calprotectin, possibly due to the low number of patients with active disease, our finding is supported by previous studies describing a positive significant correlation between Fusobacteria and clinical PDAI and CRP [18,19]. Pouchitis is most efficiently treated with antibiotics such as metronidazole and ciprofloxacin, despite the fact that not every patient will benefit from antibiotic treatment [20]. Since antibiotics are the most efficient solution, there must presumably be a link to the pouch microbiota or individual microorganisms in pouchitis. One such link, as we and others have suggested, could be a high level of Fusobacteria. Fusobacteria is a phyla of anaerobic bacteria that has been linked to both colorectal cancer and, albeit to a lesser degree, to IBD, especially UC [21]. In light of the well-known effectiveness of metronidazole for treating pouchitis, it is interesting to note that metronidazole is an effective treatment for anaerobic bacteria and that pouchitis might be linked with anaerobic Fusobacteria [22]. Furthermore, ciprofloxacin, another efficient treatment for pouchitis, might be more effective in treating Fusobacteria than Bacteroidetes, based on MIC measurements [22]. In other circumstances, the sparing of anaerobic bacteria, such as Bacteroidetes, in the gut results in a higher clinical efficacy of antibiotic drugs used for gastrointestinal infections, e.g., it has been found to be important to preserve microbiota diversity when treating *Clostridium difficile* infection (CDI), so as to reduce the risk of recurrences [23].

The exact association in pouchitis between endoscopic appearance, symptomatology as described by mPDAI, and microbiota changes is unknown. It is, however, well known that there is a poor correlation between endoscopic findings and clinical symptoms in patients with a pouch. Patients frequently describe an increase in bowel movements with no endoscopically visible inflammation [24]. In this context, other markers of inflammation, such as fecal calprotectin, are potentially more likely to indicate inflammation. Fecal calprotectin might therefore be better suited than either endoscopy or symptom scores to evaluate whether treatment with antibiotics is necessary or not. There is currently no agreed-upon calprotectin cut-off score for diagnosing pouchitis, even though it has been found that median calprotectin levels are significantly higher in patients with pouchitis than in those without pouchitis [25]. More studies are needed that investigate the relationship between fecal calprotectin, endoscopic appearance, histological findings and symptom scores with microbiota changes, both before and after successful and unsuccessful treatment of pouchitis. Our chosen fecal calprotectin cut-off of 500 mg/kg was arbitrary; however, lower calprotectin levels often seem to be associated with an absence of clinical symptoms. 

Surprisingly, and possibly in contrast to both Crohn’s disease and UC, increased Proteobacteria abundance or *E. coli* phylotypes was not found to be associated with active pouchitis in our study. This might distinguish the disease-associated microbiota in IBD from the disease-associated microbiota in pouchitis. After surgical treatment of UC, the target organ for the possible link between Proteobacteria abundance and, specifically, phylogroup B2 *E. coli* and IBD are no longer present. In this context it might not be surprising that a similar link cannot be found in pouchitis. In fact, even though we found significantly higher levels of Proteobacteria in patients with a pouch compared to healthy controls and compared to both patients with UC and CD, the level of Proteobacteria was also lower (although non-significantly) in patients with an increased inflammatory burden in their pouch based on fecal calprotectin greater than 500, as compared to patients with fecal calprotectin less than 500. Since antibiotics do fail to treat many patients with pouchitis, it is of importance that both FMT and probiotics (e.g., VSL#3) have shown promising results as treatment options for pouchitis [26,27]. However, the best bacterial composition of either a probiotic alternative to antibiotics or an ideal FMT donor for patients with pouchitis has not yet been determined. It is therefore important to identify which groups of bacteria are linked to both pouchitis and to healthy pouches. In addition, knowledge of the healthy and disease-associated pouch microbiota is important when choosing or developing new antibiotic treatments for patients with pouchitis. Identifying and focusing on the specific bacteria causing pouchitis would further enable one to look for resistance genes, possibly explaining the ineffectiveness of a previously successful antibiotic treatment.

## 4. Material and Methods

### 4.1. Population and Sample Collection

Patients from two independent studies of IBD [28,29], including fecal samples from controls and patients with UC, CD and IAPA, were included in this study, for a total of 20 patients with ileo-anal pouch anastomosis and previous flare-ups of pouchitis. Baseline data for controls and patients with UC and CD have previously been described [30]. All patients had had their pouch for more than one year, and none had used antibiotics or other anti-inflammatory drugs within the two months prior to stool sampling. For comparison, microbiota data from patients with UC and CD and from healthy controls were included in this study, the characteristics of which have previously been described [30]. All of the participants gave informed written consent. All patients were found negative in screening for bacterial (including *C. difficile* and pathogenic *E. coli*), parasitic and viral gastro-intestinal infections. Demographic and clinical characteristics of the IAPA patients are summarised in Table 1. 

### 4.2. Disease Activity and Severity

Disease activity was measured with the clinical modified pouchitis disease activity index (mPDAI) [14], a score of 3 or above indicating active disease, and with fecal calprotectin measurements, where a score of 500 or higher was taken to indicate pronounced inflammation. Endoscopy was not performed as part of the study, but based on their records, all patients with current pouchitis had previous endoscopies performed, confirming their pouchitis diagnosis. 

### 4.3. Determination of Fecal Calprotectin 

CALPRO (Calpro AS, Oslo, Norway) is an enzyme-linked immunoassay (ELISA) based on polyclonal antibodies to human calprotectin (S100A8/A9). As per the manufacturer’s instructions, 100 mg frozen fecal samples were homogenized in 4.9 mL extraction buffer using fecal extraction tubes (Calpro AS, Product No. CAL0500). The supernatants were diluted in sample diluent solution (1:100) before testing [31].

### 4.4. Isolation of E. coli from Fecal Samples

Fecal samples were sent for analysis at Statens Serum Institut, Copenhagen, Denmark. Ten μg fresh stool sample was mixed in 2 mL phosphate buffered saline (PBS, pH 7.38) and 10 μL was plated on SSI enteric medium agar plates (SSI, Hillerød, Denmark, product no. 724) and incubated overnight at 37 °C. Bacteria from the SSI enteric medium were harvested and plated on SSI blue agar plates (SSI, Hillerød, Denmark, product no. 694) selective for gram negative bacteria and incubated overnight at 37 °C. *E. coli* colonies were isolated from the SSI blue agar plate and tested for Beta-glucuronidase40 ((PGUA), SSI, Hillerød, Denmark, product no. 1033) and Indol (Biomérieux, Denmark, product no. 56541). Isolated *E. coli* were inoculated in Luria broth (LB) (Sigma-Aldrich, GmbH, Germany) and incubated overnight at 37 °C. Twenty-five μL of bacterial culture in LB were diluted in 975 μL of sterile water, boiled at 100 °C for 15 min, centrifuged for 10 minutes at 14,000× *g*, and the supernatant (bacterial DNA) was transferred to a new tube and stored at −20 °C for PCR testing. *E. coli* isolates with different colony morphologies from the SSI blue agar plates were harvested, dissolved in 15% glycerol (Sigma-Aldrich, GmbH, Germany. Cat: 67757) in beef stock (SSI, Hillerød, Denmark, product no. 1056), and stored at −80 °C.

### 4.5. E. coli Phylogenetic Group Determination

*E. coli* phylogenetic groups (A, B1, 2 and D) were determined by a simple PCR procedure based on the genes chuA, yjaA and an anonymous DNA fragment, using primers and conditions exactly as described by Clermont et al. [32] We have previously found that this simple triplex PCR procedure separates IBD associated *E. coli* from *E. coli* from other sources and from *E. coli* from patients with inactive disease, as well as more complex typing methods [33,34]. 

### 4.6. DNA Extraction for Microbiota Analysis 

DNA was extracted using the PowerSoil DNA Isolation Kit (MO-BIO), which has previously been found to be well-suited for fecal samples [35]. Negative controls were included from DNA extraction [36]. All plates included a mock community to serve as an internal control for calibration of the bioinformatical pipeline. 

The 16S rDNA V3-V4 region was targeted using the primers S-D-Bact-0341-b-S-17 and S-D-Bact-0785-a-A-21 [37]. The following PCR program was used: 98 °C for 30 sec, 25x (98 °C for 10 s, 55 °C for 20 s, 72 °C for 20 s) and 72 °C for 5 min. Amplification was verified by running the products on an agarose gel. Indices were added in a subsequent PCR using the Nextera Index Kit V2 (Illumina), with the following PCR program: 98 °C for 30 sec, 8x (98 °C for 10 s, 55 °C for 20 s, 72 °C for 20 s) and 72 °C for 5 min. The attachment of barcodes was verified by running the products on an agarose gel.

Products from the nested PCR were pooled and the resulting library was cleaned with the Agencourt AMPure XP PCR purification kit (Beckman Coulter). The DNA concentration of pooled libraries was measured on a Qubit fluorometer using the Qubit High Sensitivity Assay Kit (Thermo Fisher Scientific). Sequencing was done on an Illumina MiSeq desktop sequencer using the MiSeq Reagent Kit V3 (Illumina) for 2x 300 bp paired-end sequencing. 

### 4.7. Bioinformatical Analysis

The 64-bit versions of USEARCH [38] and mothur [39] were used in combination with several in-house programs for a bioinformatical analysis of the sequence data. Following tag identification and trimming, all sequences from all samples were pooled. Paired-end reads were merged, truncating reads at a quality score of 4, requiring at least 100 bp overlap and a merged read length of between 300 and 600 bp. Sequences with ambiguous bases, without perfect match to the primers, or homopolymer length greater than 8 were discarded and primer sequences trimmed. Reads were quality filtered, discarding reads with more than 5 expected errors and sequences were strictly dereplicated, discarding clusters smaller than 5. Sequences were clustered into operational taxonomical units (OTUs) at 97% sequence similarity, using the most abundant strictly dereplicated reads as centroids and discarding suspected chimeras based on internal comparison. Additional suspected chimeric OTUs were discarded based on comparison with the Ribosomal Database Project classifier training set v9 using UCHIME [40]. Taxonomic assignment of OTUs was carried out using the method of Wang et al. [41], with mothur’s PDS version of the RDP training database v14. Following this, samples were rarified to the lowest sequence number found in a sample (2780).

### 4.8. Ethical Considerations

Permission for the study was obtained from the Regional Ethics Committee for Copenhagen County Hospitals (Permission no. KA-20060159 and H-1-2011-088). Permission was also obtained from the Danish Data Registry (01769 HVH-2012-027).

## 5. Conclusions

In conclusion, we found a high level of Fusobacteria colonization among patients with Ileo-anal pouch anastomosis and marked pouch inflammation. This link between Fusobacteria and pouch inflammation could possibly be significant for future diagnostics and treatment options of pouchitis. Even though, patients with Ileo-anal pouch anastomosis had an increased abundance of Proteobacteria colonization compared to patients with ulcerative colitis or Crohn’s disease and healthy controls, no significant link between pouch inflammation and Proteobacteria or other bacterial phyla or *E. coli* phylogroups could be demonstrated

## Figures and Tables

**Table 1:** Patient characteristics, modified pouchitis disease activity index (mPDAI), calprotectin, *E. coli* phylogroup, relative sequence abundance of Proteobacteria, Shannon index and number of operational taxonomical units (OTUs). Bold: active disease, mPDAI > 2

Patient ID.	Age	Sex	MPDAI	Calprotectin	*E. coli* Phylogroup	Abundance of Proteobacteria (%)	Shannon Index	Number of OTUs
**P17**	**24**	**F**	**6**	**2009**	**A**	**3.71**	**1.53**	**39**
**P20**	**38**	**F**	**4**	**62**	**B1, A**	**36.33**	**1.53**	**29**
**P21**	**25**	**M**	**5**	**550**	**B2, A**	**8.53**	**2.08**	**45**
**P25**	**47**	**M**	**4**	**80**	**B1**	**1.51**	**1.61**	**67**
**P47**	**32**	**M**	**3**	**111**	**D**	**88.17**	**0.51**	**22**
**P68**	**42**	**F**	**5**	**1707**	**A**	**98.71**	**0.08**	**7**
**P71**	**79**	**F**	**4**	**<50**	**no *E. coli***	**42.34**	**1.99**	**35**
**P74**	**27**	**M**	**6**	**2309**	**no *E. coli***	**4.1**	**2.84**	**125**
**P80**	**60**	**M**	**3**	**296**	**D**	**9.75**	**2.95**	**87**
**P121**	**62**	**F**	**6**	**111**	**B2**	**0.18**	**2.46**	**67**
P1	40	M	0	71	A	19.68	3.51	147
P58	41	F	1	163	A	49.64	1.40	26
P62	50	M	0	NA	no *E. coli*	0.22	1.08	22
P63	35	M	0	<50	D, B2	88.71	0.69	18
P94	44	F	0	260	no *E. coli*	0.90	2.33	50
P97	32	M	0	<50	no *E. coli*	0.61	3.00	90
P104	57	M	1	<50	B2, A, D	4.06	2.16	45
P130	40	F	0	119	A	51.08	1.45	28
P150	37	M	1	1132	B2, B1	2.19	2.38	59
P200	36	F	2	1566	no *E. coli*	2.84	2.03	38
